# Massachusetts Veterinary Association

**Published:** 1885-10

**Authors:** L. H. Howard

**Affiliations:** Secretary


### MASSACHUSETTS VETERINARY ASSOCIATION.

The regular meeting was held at 19 Boylston Place, Boston, Wednesday evening, June 3d.

In the absence of the President, Vice-President J. S. Saunders presided, and there were present thirteen members, viz: Drs. Alderman, Blackwood, Bryden, Bunker, Harrison, Howard, Osgood, J. S. Saunders, F. Saunders, Simmons, Skally, Winchester and Winslow.

Inquiry being made as to progress of Committee on Charter, they reported that owing to the close of session of the Legislature no progress could be made at present.

Some discussion taking place as to the legal points of the subject, Dr. Osgood moved that the committe be authorized to consult a lawyer, and Dr. Winchester was accordingly authorized to gain legal information, he having already approached Attorney-General Sherman in regard to the matter.

The committee appointed at the May meeting, to prepare resolutions censuring the pursuance of the so-called "subscription plan" by veterinary institutions, reported the following:

"WHEREAS, we, the Massachusetts Veterinary Association, a body composed of regularly graduated veterinarians, having for its objects the elevation and advancement of the veterinary profession and the science of veterinary medicine, and the encouragement of collegiate and professional feelings among the members of the profession practicing in the State of Massachusetts ; and

"WHEREAS, we believe such objects are to be obtained only by each member of the profession as an individual, and each organization as a body, using its every effort to that end, commending and applauding whatever advances and honors the cause, and discouraging and condemning that which dishonors it and retards its progress ; and

"WHEREAS, a system of business management pursued by different veterinary institutions in this country and England, and known as the 'subscription plan,' has, in the experience of our *confréres*, been derogatory and detrimental to the best interests of the science and profession ; and

"WHEREAS, we, the members of the Massachusetts Veterinary Association, have realized by our own experience that the said 'subscription plan,' as pursued by the management of the Harvard Veterinary Hospital in this city and State, is *repeating* its disgraceful history, in exerting a most injurious influence upon members of the profession practising in the State of Massachusetts, is already threatening defeat to the objects of our Association, and is thereby a foe to the cause of veterinary science and the veterinary profession ; be it therefore

"*Resolved*, that we, the members of the Massachusetts Veterinary Association, in meeting assembled, do hereby, as a body, express our disapproval and condemnation of the adoption and continued pursuance by the management of the Harvard Veterinary Hospital of the said 'subscription plan ;' and be it

*Resolved*, that it is the duty of every member of this Association to use his utmost influence to have said plan discontinued and abolished."

J. S. SAUNDERS, D.V.S.,
MADISON BUNKER, D.V.S., } *Committee.*
L. H. HOWARD, D.V.S.,

Following the reading of the above report quite a lengthy discussion of the question took place, which called forth a very general expression of opinion.

It was finally voted to accept the report, by a nearly unanimous vote.

Dr. Bryden presented the name of Charles M. Bailey, V.S., of Haverhill, as an applicant for membership, which was referred to the Executive Committee.

Dr. H. L. Alderman read a paper on "Laminitis in the Horse."

He first remarked that the disease was not confined to the feet, but that the whole organism was affected, and advised that in diseases of the lungs, in heavy horses particularly, the feet to be poulticed to prevent metastatic laminitis.

The essayist gave a very clear description of the course of laminitis, and the different forms of treatment, and the paper was provocative of a good discussion as to causes operating to produce laminitis, different modes of treating it, including various ways of shoeing, to afford relief, etc.

At its conclusion a unanimous vote of thanks was tendered the essayist.

No other business coming before the meeting, it was adjourned.

L. H. HOWARD, Secretary.

The regular monthly meeting of the Massachusetts Veterinary Association was held in Boston, Wednesday evening, July 1st.

Dr. Billings presided, and there were also present Drs. Blackwood, Bryden, Byrne, Howard, Peters, J. S. Saunders, Skally, Winchester and Winslow.

The first part of the evening was taken up by a discussion in regard to the duties of the Executive Committee, and a report of progress by the committee on Charter.

The meeting then listened to Part I. of an essay on "The Horse's Foot," by Dr. Bryden, the essayist promising to carry out his original intention of a further treatment of this subject.

He first called attention to the importance of study as to conditions of the horse's foot, even from the time of its first formation during fœtal life, as well as the conditions affecting its growth afterward, such as locality, climate, soil, etc.

He said, we have different varieties of hoof, according to the locality in which the horse is raised, and according to what its surroundings are.

He called attention to the necessity of guarding against the changes which domestication produces, and explained how an irregularly growing hoof would cause distortion and unsymmetrical development of all tissues above, producing spavins, ringbones, bent hocks, dimpled hips, tail drawn to one side, etc , thus affecting not only the action, but shape as well, of the animal.

At the conclusion of his remarks a vote of thanks was tendered the essayist, and he was asked to favor us at a later day with his further treatment of the subject.

Dr. Peters was appointed essayist for the September meeting, and the company adjourned.

L. H. HOWARD, Secretary.

